# Supplementary material for: Rate of reclassification of HER2-equivocal breast cancer cases to HER2-negative per the 2018 ASCO/CAP guidelines and response of HER2-equivocal cases to anti-HER2 therapy
Source: PLoS One. 2020 Nov 12;15(11):e0241775. doi: 10.1371/journal.pone.0241775 (PMC7660495; doi:10.1371/journal.pone.0241775)
Supplement: S1 Table — (DOCX) [file pone.0241775.s001.docx]

**S1 Table. Backbone chemotherapy regimens containing anti-HER2 therapy.**

| Regimen | | No. of patients |
| --- | --- | --- |
| Primary cohort |  |  |
| Neoadjuvant regimen |  |  |
|  | AC/THP | 2 |
|  | TCHP/AC | 1 |
| Adjuvant regimen |  |  |
|  | TCH | 1 |
|  | AC/TH | 1 |
| Recurrent/metastatic cohort |  |  |
|  | PTH | 1 |

AC/THP: doxorubicin, cyclophosphamide, paclitaxel, trastuzumab, and pertuzumab. TCHP/AC: docetaxel, carboplatin, trastuzumab, pertuzumab, doxorubicin, and cyclophosphamide. TCH: docetaxel, carboplatin, and trastuzumab. AC/TH: doxorubicin, cyclophosphamide, paclitaxel, and trastuzumab. PTH: paclitaxel, trastuzumab, and pertuzumab.
